# Habituation in the Tail Withdrawal Reflex Circuit is Impaired During Aging in *Aplysia californica*

**DOI:** 10.3389/fnagi.2016.00024

**Published:** 2016-02-12

**Authors:** Andrew T. Kempsell, Lynne A. Fieber

**Affiliations:** Division of Marine Biology and Ecology, Rosenstiel School of Marine and Atmospheric Science, University of MiamiMiami, FL, USA

**Keywords:** short term memory, long term potentiation, pleural ganglion, pedal ganglion, marine invertebrate

## Abstract

The relevance of putative contributors to age-related memory loss are poorly understood. The tail withdrawal circuit of the sea hare, a straightforward neural model, was used to investigate the aging characteristics of rudimentary learning. The simplicity of this neuronal circuit permits attribution of declines in the function of specific neurons to aging declines. Memory was impaired in advanced age animals compared to their performance at the peak of sexual maturity, with habituation training failing to attenuate the tail withdrawal response or to reduce tail motoneuron excitability, as occurred in peak maturity siblings. Baseline motoneuron excitability of aged animals was significantly lower, perhaps contributing to a smaller scope for attenuation. Conduction velocity in afferent fibers to tail sensory neurons (SN) decreased during aging. The findings suggest that age-related changes in tail sensory and motor neurons result in deterioration of a simple form of learning in *Aplysia*.

## Introduction

A significant portion of research in the neurobiology of aging is focused on identifying age-related changes in learning and memory. Mammalian models have focused attention on the molecular and cellular processes at risk in aging (Kumar and Foster, 2007). Nervous system aging is associated with declines in synaptic efficacy and neuronal excitability that contribute to memory loss (Rogers et al., 1981; Barnes, 1990; Turner and Deupree, 1991; Barnes et al., 1992, 2000; Foster and Norris, 1997; Jouvenceau et al., 1998; Oh et al., 2010; Kumar et al., 2012). The number and size of synapses decreased during aging in several neuronal types (Foster, 2002). Conduction velocity decreased in neuronal processes of neocortical, cerebellar, and peripheral sensory neurons (SN) and motoneurons (MN; Rogers et al., 1981; Aston-Jones et al., 1985; Chase et al., 1985; Lamour et al., 1985; Kanda et al., 1986; Morales et al., 1987; Boxer et al., 1988; Xi et al., 1999). Neuronal excitability, measured as the threshold to elicit action potentials (AP) by intracellular current injection, was reduced in the hippocampus and cerebellum of aged animals (Rogers et al., 1981; Turner and Deupree, 1991). Aging of hippocampal neurons resulted in an increase in the amplitude of the Ca^2+^-dependent, K^+^-mediated afterhyperpolarization (Landfield and Pitler, 1984; Kerr et al., 1989; Disterhoft et al., 1996; Hsu et al., 2002), resulting in altered synaptic function and learning in aged animals (Kumar and Foster, 2002; Bodhinathan et al., 2010; Kumar et al., 2012).

The complexity of the mammalian brain makes it difficult to study aging in defined circuits and individual neurons, particularly when trying to couple changes at the neuronal level with changes in even the simplest of behaviors. Age-related neural changes in *Aplysia californica* (*Aplysia*), such as declines in sensory and motoneuron excitability (Kempsell and Fieber, 2014, 2015b) and failure to respond to second messengers (Kempsell and Fieber, 2015a), mirror hallmarks of aging in vertebrates, but are easily studied in this model organism. Simple behaviors in *Aplysia*, such as habituation, can be studied in individual neurons of simple reflex circuits to better understand aging-associated effects.

This study used a simple neural circuit of *Aplysia*, the tail withdrawal reflex (TWR), to investigate nervous system aging with an emphasis on habituation. Direct stimulation of the tail initiates TWR, and involves identified SN in the pleural ganglia and MN in the pedal ganglia (Walters et al., 1983a, 2004). Distinct forms of nonassociative learning, including habituation, dishabituation, and sensitization, can be evoked in TWR. Habituation in TWR is a decrease in reflexive response after repeated application of an innocuous stimulus. These changes in behavior are reflected in concurrent changes in the efficacy between tail SN and MN synapses. For example, homosynaptic depression is induced during habituation.

We have recently described changes in short-term memory for sensitization in TWR and related declines in facilitation between tail SN-MN synapses during aging, suggesting that nonassociative learning is impaired in aged *Aplysia* (Kempsell and Fieber, 2015a, b). This study addressed another form of nonassociative learning in *Aplysia*. We measured habituation in TWR and related depression in tail MN during aging. Conduction velocity in nerve P9 innervating TWR was assessed. We found that habituation in TWR was impaired in aged animals, and that the neural circuit for TWR was also compromised.

## Materials and Methods

Cohorts of *Aplysia* from the University of Miami National Resource for *Aplysia* were reared and used in experiments as described below, and previously (Kempsell and Fieber, 2015a).

Behavioral and electrophysiological experiments were executed on sibling animals when mature and at aged II, as described in Kempsell and Fieber (2014). Mature animals were age 7–8 months and sexually reproductive for ≥1 month. Aged II animals were age 12–13 months, and had reduced performance in the righting reflex, TWR, and biting response compared to mature and aged I siblings (Kempsell and Fieber, 2014). Although approximately 1 month from end of life, and displaying impaired reflex times, aged II animals sought out food, although with reduced appetite, and thus had reduced mass, since they continued to reproduce.

### Habituation of TWR in Freely Behaving *Aplysia*

Animals were placed individually in 48 × 27 × 20 cm translucent plastic cages filled with 20°C seawater to a depth of 15 cm and acclimated for 5 min. A trained experimenter unaware of the age of the animals conducted behavioral measurements.

The same 12 animals from a single cohort were measured for habituation in TWR at mature and aged II time points following previously defined protocols (Stopfer and Carew, 1996; Watkins et al., 2010; Kempsell and Fieber, 2014). Each animal was placed on its foot in the center of the cage and allowed to acclimate for 5 min. The animal’s resting body length was measured in cm. A 500 ms tap to the tip of the tail at a stimulus pressure of 75 g/mm^2^ initiated tail withdrawal. The retracted body length was measured, and, expressed as a fraction of resting length, was designated TWR amplitude. Thirty percent relaxation to resting tail length signified the end of the reflex; the time this took was designated TWR duration. TWR amplitude and TWR duration were measured at 15, 10, and 5 min before habituation training (−15, −10, −5 min) and the average of these three measurements was designated baseline. Habituation training proceeded, consisting of 30 trials of 500 ms tail taps at a stimulus pressure of 75 g/mm^2^, with an interstimulus interval (ISI) of 30 s delivered to the tip of the tail at the same site as during baseline measurements and post-training tests (post-test). TWR was again elicited, as post-tests, 5, 10, and 15 min following habituation training (time points: 20, 25, 30 min), and amplitude and duration were measured.

### Intracellular Recording of TWR in Semi-Intact Tail Preparation

In electrophysiological experiments investigating SN and MN response to tail stimulation, a ganglia-tail preparation was used that consisted of the left or right pleural-pedal hemiganglia connected to the tail by nerve P9 (Kempsell and Fieber, 2015b), with all other connectives severed. The same cohort of animals was studied at maturity and aged II. Animals were anesthetized by injecting isotonic MgCl_2_ (~50% animal weight by volume) into the body cavity. Pleural-pedal ganglia, nerve P9, and attached tail were removed and pinned tightly to a Sylgarded dish. The remaining ganglia were removed from the body cavity to euthanize the animal, and these unneeded ganglia and tissues were discarded. The pins were positioned in the reduced tail preparation to minimize tail contraction following mechanical and electrical stimulation. Ganglia were surgically desheathed and were bathed during the experiment in flowing artificial seawater (ASW) via a gravity-fed perfusion pipette within ~5 mm of the ganglia. Tail SN of the ventral caudal region of the pleural ganglia (PVC) were not spontaneously active and had resting potentials of −40 to −55 mV, but produced AP of 60–100 mV amplitude when receptive fields on the tail were stimulated (Walters et al., 2004). Tail pedal MN P5–7 were spontaneously active with resting potentials of −40 to −70 mV and exhibited increased AP firing in response to mechanical stimulation of the tail (Walters et al., 1983b).

Glass capillary microelectrodes of 5–15 MΩ resistance were used for intracellular recordings in tail SN and MN. All recordings were made using pClamp10 software with BRAMP-01R and ELC-01MX amplifiers (ALA Scientific Instruments, Farmingdale, NY, USA) connected to a PC and Digidata 1440A A/D converter.

Tail MN responses to mechanical tail stimulation were monitored before, during, and after habituation training in intact tail-ganglia preparations to determine aging effects. Three pre-training tests were recorded and the baseline response was calculated from their average. Habituation training consisted of 30 tail taps at 30 s ISI (same as described above for behavioral experiment in intact animals). Post-test responses were then evoked at 5, 10, and 15 min after training.

For conduction velocity experiments, weak tail shock (~3–6 nA) sufficient to evoke 1–5 AP was applied to the mechanosensory field of tail SN. The latency between tail shock and initiation of the first evoked AP was measured in mature and aged II preparations. The distance between SN somata and the position of shock delivery on the tail was measured with calipers and used to calculate the approximate conduction velocity in nerve P9.

### Solutions

Extracellular solution consisted of ASW containing (mM) 417 NaCl, 10 KCl, 10 CaCl_2_, 55 MgCl_2_, and 15 HEPES-NaOH, pH 7.6. The pipette solution for intracellular recordings in intact ganglia consisted of 3 M KCl. All reagents were from Sigma-Aldrich (St. Louis, MO, USA).

### Data Acquisition and Analysis

Data were expressed as mean ± SE. Significant differences, such as that occurring during habituation training, were determined by one-way within subjects (repeated-measures) ANOVA. After the ANOVA, individual differences were compared against baseline using Tukey’s *post hoc* test. Comparisons of mature and aged II responses were made using 2-sample *t*-tests. All analyses were performed using the open source R statistical program (Vienna, Austria). Differences at *p* ≤ 0.05 were accepted as significant.

## Results

The median lifespans of the two cohorts studied were 362 and 369 days. Animals were sexually mature by age 7 months. Morphological and aging characteristics were as described in a previous study (Kempsell and Fieber, 2014).

### Habituation Training in Intact Animals Reduced TWR in Mature but not Aged II *Aplysia*

**Figure 1:**
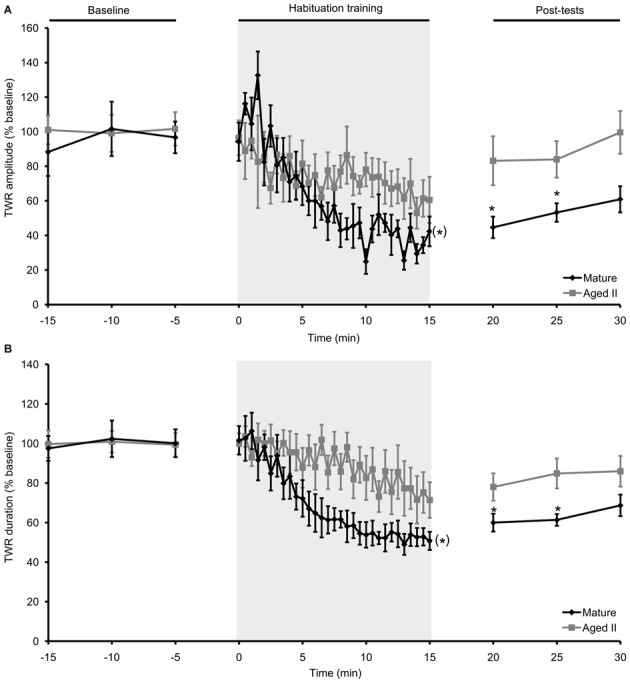
**Habituation in the TWR was disrupted during aging. (A)** In mature animals, TWR amplitude decreased significantly during habituation training (30 trials of 500 ms, 75 g/mm^2^ tail tap; 30 s ISI) and remained habituated at 5 and 10 min post-tests. In the same animals at stage aged II, training did not change TWR amplitude during training or in post-tests. **(B)** TWR duration decreased significantly during habituation training in mature animals and remained habituated at 5 and 10 min post-tests. When tested at aged II, training had no effect on TWR duration. ^(*)^Denotes significant decrease during habituation training in mature at *p* ≤ 0.05 via repeated measures ANOVA; *Denotes significant difference in post-test compared to baseline at *p* ≤ 0.05 via Tukey’s *post hoc* tests (*n* = 12).

In our previous studies (Kempsell and Fieber, 2014, 2015a,b), we established that baseline TWR amplitude in these cohorts was significantly weaker (mature: 8.0 ± 0.3% body withdrawn; aged II: 6.8 ± 0.5% body withdrawn; *p* ≤ 0.01, 2-sample *t*-test) and TWR duration was significantly slower (mature: 14.0 ± 1.2 s; aged II: 22.9 ± 1.6 s; *p* ≤ 0.01, 2-sample *t*-test) at stage aged II compared to mature. Prior studies in *Aplysia* demonstrated that habituation training results in decreased TWR amplitude and duration (Carew et al., 1972; Stopfer and Carew, 1996), We confirmed this with habituation training in mature hatchery animals, where tail withdrawal decreased significantly in amplitude (Figure 1A, *p* ≤ 0.05, repeated measures ANOVA) and duration (Figure 1B, *p* ≤ 0.05, repeated measures ANOVA). TWR amplitude and duration were significantly reduced below baseline by trial 14 and 15 of habituation training, respectively, and these measures remained significantly reduced for the rest of training (*p* ≤ 0.05 for trials 14–30 and 15–30 compared to baseline, Tukey’s *post hoc* tests). TWR amplitude and duration remained significantly reduced from baseline for up to 10 min after the conclusion of training (TWR amplitude: *p* ≤ 0.01 at 5 min post-test compared to baseline, *p* ≤ 0.05 at 10 min; TWR duration: *p* ≤ 0.05 at 5 and 10 min post-test compared to baseline; Tukey’s *post hoc* tests). Aging significantly affected habituation. In the same animals tested when they reached stage aged II, TWR amplitude and duration did not change significantly from baseline during habituation training or in post-tests. Thus, short forms (≤30 min) of memory for habituation in TWR were impaired in aged II *Aplysia*.

### Habituation Training in Reduced Ganglia-Tail Preparations Decreased Responses in Mature but not Aged II Tail MN

Mean resting potentials and mean input resistances of tail SN or tail MN did not change between maturity and aged II and were as previously reported (resting membrane potential: −45.8 ± 4.2 mV for mature SN, −44.9 ± 6.2 mV for aged II SN, −54.3 ± 5.3 mV for mature MN, −53.2 ± 10.4 mV for aged II MN; input resistance: 26.1 ± 5.1 MΩ for mature SN, 25.3 ± 4.6 MΩ for aged II SN; 10.5 ± 2.1 MΩ for mature MN, 9.9 ± 2.7 MΩ for aged II MN; *p* > 0.05 in each case; 2-sample *t*-test; Kempsell and Fieber, 2014, 2015b). AP duration (mature: 3.8 ± 0.6 ms, aged II: 4.0 ± 0.9 ms) and depolarization amplitude (mature: 88 ± 11 mV, aged II: 82 ± 13 mV) were not different between mature and aged II SN. AHP amplitude in tail SN also was not different (mature: 4.2 ± 0.7 mV, aged II: 4.7 ± 0.9 mV), as previously reported (Kempsell and Fieber, 2014, 2015b).

**Figure 2:**
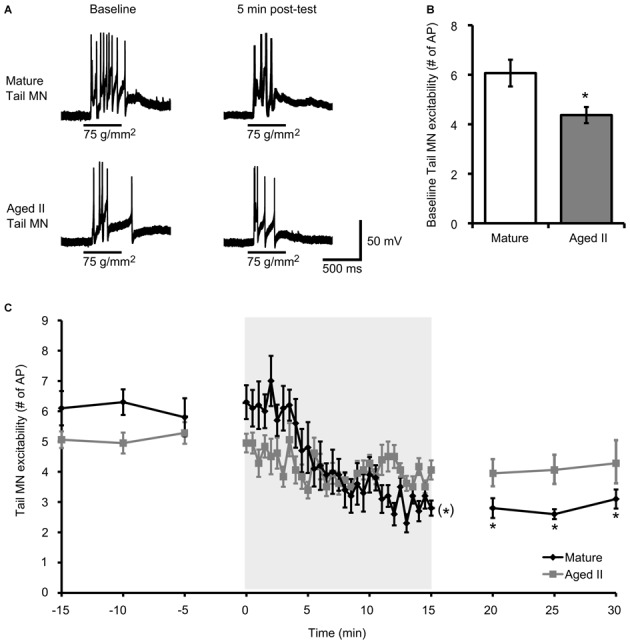
**Aging affects tail MN response following habituation training. (A)** A 500 ms tap to the tail at a stimulus pressure of 75 g/mm^2^ evoked AP in tail MN from mature and aged II reduced tail preparations. Tail MNs were hyperpolarized with just enough current to suppress spontaneous activity (average holding potential: −64 ± 4.1 mV for mature, −63 ± 7.8 mV for aged II). Training in mature preparations reduced response to tail tap, but aged II preparations were unaffected, as summarized in **(C). (B)** Baseline excitability of tail MN to tail tap decreased significantly during aging. *Denotes significant decrease compared to mature at *p* ≤ 0.05, 2-sample *t*-test. **(C)** Following habituation training (30 trials of 500 ms, 75 g/mm^2^ tail tap; 30 s ISI), mature tail MN fired fewer AP in post-tests compared to baseline. ^(*)^Denotes significant decrease during habituation training in mature at *p* ≤ 0.05 via repeated measures ANOVA; *Denotes significant decrease in mature post-test compared to baseline at *p* ≤ 0.05 via Tukey’s *post hoc* tests (*n* = 10). In aged II preparations, no significant habituation occurred (*p* = 0.08; *n* = 9).

Habituation training was demonstrated to decrease excitability in *Aplysia* MN (Castellucci et al., 1978; Stopfer and Carew, 1996; Stopfer et al., 1996; Glanzman, 2009). To determine whether aging affected this phenomenon, MN excitability, quantified as the number of AP fired during 500 ms tail tap, was evaluated in mature and aged II reduced tail preparations before and after habituation training (Figure 2A). Baseline MN response to tail tap decreased significantly between mature and aged II stages (Figure 2B, *p* ≤ 0.01; 2-sample *t*-test), as demonstrated previously (Kempsell and Fieber, 2015b), confirming that aged II MN excitability decreased with aging. During habituation training, tail MN excitability decreased significantly in preparations from mature animals (Figure 2C, *p* ≤ 0.05, repeated measures ANOVA). Tail MN excitability was significantly reduced below baseline by trial 17 of habituation training and remained significantly reduced for the rest of training (*p* ≤ 0.05 for trials 17–30 compared to baseline, Tukey’s *post hoc* tests) and for 5, 10, and 15 min after the conclusion of training (*p* ≤ 0.05 at each post-test compared to baseline, Tukey’s *post hoc* tests). In contrast, no further changes in aged II tail MN excitability were found during habituation training or in post-tests (*F*_(29,290)_ = 1.31, *p* = 0.08).

### Conduction Velocity Between Tail and SN Soma Decreased During Aging

**Figure 3:**
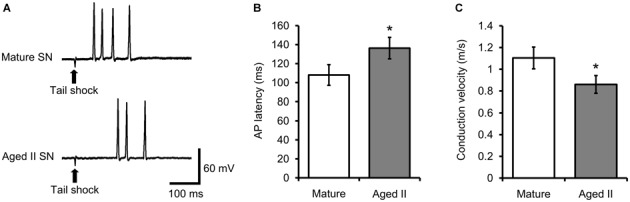
**Conduction velocity decreased during aging in tail SN. (A)** Weak tail shock (3–6 nA, 3 ms) in mechanosensory field of innervating tail SN evoked 1–5 AP in tail SN, with latency summarized in **(B). (B)** The latency between tail shock and initiation of the first evoked AP increased significantly in aged II. *Denotes significant difference from mature at *p* ≤ 0.05, 2-sample *t*-test. **(C)** Conduction velocity in nerve P9 decreased significantly during aging. *Denotes significant difference from mature at *p* ≤ 0.05, 2-sample *t*-test (*n* = 17 for mature, *n* = 21 for aged II).

Studies in vertebrate models have shown that conduction velocity, the speed at which AP travel down the axon, decreased during aging in processes of neocortical, cerebellar, and peripheral sensory and motor neurons (Rogers et al., 1981; Aston-Jones et al., 1985; Chase et al., 1985; Lamour et al., 1985; Kanda et al., 1986; Morales et al., 1987; Boxer et al., 1988; Xi et al., 1999). We investigated whether conduction velocity between mechanoreceptive field on the tail and SN soma changed during aging by measuring the latency between tail shock and AP discharge in tail SN. Weak tail shock to the mechanosensory field of tail SN evoked 1–5 APs in mature and aged II reduced preparations (Figure 3A). The latency between tail shock and initiation of the first evoked AP increased significantly between mature and aged II preparations (Figure 3B, *p* ≤ 0.05; 2-sample *t*-test). Conduction velocity was 1.1 ± 0.11 m/s in mature and 0.86 ± 0.10 m/s in aged II, a significant difference (Figure 3C, *p* ≤ 0.05; 2-sample *t*-test).

## Discussion

This study focused on age-related memory loss in the *Aplysia* model that builds on previous studies demonstrating that a SN-MN circuit such as for TWR is useful for understanding aging mechanisms in the nervous system. Failure to habituate in aged animals is a part of a continuum of aging declines noted in the intact TWR that coincide with concomitant changes in tail SN and MN excitability compared to that of younger sibling animals (Fieber et al., 2010; Kempsell and Fieber, 2014, 2015a,b; Dunn and Sossin, 2015). The TWR of intact animals declined in amplitude and slowed in duration during aging, and had related neural correlates. Baseline tail MN excitability was significantly lower in aged animals, both in our prior work and in these experiments, as reported in other animals (Landfield and Pitler, 1984; Kerr et al., 1989; Disterhoft et al., 1996; Foster, 2002; Oh et al., 2010). This decreased baseline excitability may provide the explanation for habituation failure of TWR, because it might mean that the scope for further synaptic depression in tail MN that should have occurred during habituation was already reached during aging, and could not be further reduced. The failure of habituation training to induce synaptic depression in aged tail MN also suggests impairment of modulation of the TWR neural circuit responsible for habituation. Conduction velocity in the tail nerve decreased, suggesting that aging negatively affects multiple aspects of tail SN-MN synaptic transmission. Decreased excitability of both SN and MN as well as reduced transmission between them may underlie the age-related deficits in learning and memory observed in this study and others (Kempsell and Fieber, 2015b).

The absence of habituation in TWR in aged animals suggested that at least some forms of nonassociative learning are compromised during aging. Habituation failure in aged *Aplysia* correlates well with other aging studies in this organism that found changes in habituation of the gill-siphon withdrawal reflex and changes in forms of short-term memory for sensitization in TWR (Rattan and Peretz, 1981; Kempsell and Fieber, 2015b). It was recently reported that cultured SN of aged *Aplysia* that form synaptic connections with MN have both reduced recovery from synaptic depression and reduced protein kinase C activation after exogenous serotonin (Dunn and Sossin, 2015), providing additional evidence of age-related changes in the TWR circuit responsible for habituation of the reflex. Furthermore, two studies that found that protein kinase activation rescued performance of aged SN and MN in the TWR circuit (Dunn and Sossin, 2015; Kempsell and Fieber, 2015a), suggested age-related impairment of the signal transduction pathway upstream of protein kinase activation.

Conduction velocity in afferent fibers decreased in aged animals, consistent with mammalian studies indicating reduced conduction velocity in processes of neocortical, cerebellar, and peripheral sensory and motor neurons (Barnes et al., 1992, 1997; Jouvenceau et al., 1998; Potier et al., 2000). This increase in the latency of transmission between mechanoreceptive fields on the tail and tail SN may cause impairments in the temporal processing of sensory information during aging, contributing to declines in memory for habituation.

The parallels between *Aplysia* and vertebrate neurophysiologies support the prospect that *Aplysia* aging studies may identify molecular targets with therapeutic potential in age-related memory failure. The connection between behavior and the cellular communication accessible by studies of membrane excitability and receptor physiology is most direct in animal models with simple brains. Our results of the failure of synaptic depression in the same aged animals that showed behavioral declines demonstrate the *Aplysia* model’s use in connecting individual neurons and synapses more directly to behavioral aging than is possible in vertebrate models.

## Conclusion

Habituation training failed to induce behavioral habituation or related depression of tail MN excitability in aged sea hares, suggesting that this form of nonassociative learning is compromised in aged *Aplysia*, as in other animal models (Fraley and Springer, 1981; Leussis and Bolivar, 2006). Loss of modulation of the TWR circuit in aged animals was correlated with failure to habituate.

## Author Contributions

The study was conceived by LAF and ATK. The study was executed by ATK. The data were analyzed, conclusions drawn and manuscript written by ATK and LAF.

## Funding

This work was funded by National Institutes of Health Grant P40 OD010952, and Maytag, Koczy, and Korein Foundation fellowships to ATK.

## Conflict of Interest Statement

The authors declare that the research was conducted in the absence of any commercial or financial relationships that could be construed as a potential conflict of interest.

## Abbreviations

TWR: tail-elicited tail withdrawal reflex
SN: sensory neuron
MN: motoneuron
PVC: pleural ventral caudal
L-Glu: L-glutamate
AP: action potential.
